# Peptide-based approaches to directly target alpha-synuclein in Parkinson’s disease

**DOI:** 10.1186/s13024-023-00675-8

**Published:** 2023-11-09

**Authors:** Scott G. Allen, Richard M. Meade, Lucy L. White Stenner, Jody M. Mason

**Affiliations:** Department of Life Sciences, University of Bath, Claverton Down, Bath BA2 7AY UK

**Keywords:** Alpha-Synuclein, Synucleinopathies, Parkinson’s Disease, Peptide therapeutics, Peptidomimetics

## Abstract

Peptides and their mimetics are increasingly recognised as drug-like molecules, particularly for intracellular protein-protein interactions too large for inhibition by small molecules, and inaccessible to larger biologics. In the past two decades, evidence associating the misfolding and aggregation of alpha-synuclein strongly implicates this protein in disease onset and progression of Parkinson’s disease and related synucleinopathies. The subsequent formation of toxic, intracellular, Lewy body deposits, in which alpha-synuclein is a major component, is a key diagnostic hallmark of the disease. To reach their therapeutic site of action, peptides must both cross the blood-brain barrier and enter dopaminergic neurons to prevent the formation of these intracellular inclusions. In this review, we describe and summarise the current efforts made in the development of peptides and their mimetics to directly engage with alpha-synuclein with the intention of modulating aggregation, and importantly, toxicity. This is a rapidly expanding field with great socioeconomic impact; these molecules harbour significant promise as therapeutics, or as early biomarkers during prodromal disease stages, or both. As these are age-dependent conditions, an increasing global life expectancy means disease prevalence is rising. No current treatments exist to either prevent or slow disease progression. It is therefore crucial that drugs are developed for these conditions before health care and social care capacities become overrun.

Parkinson’s disease (PD) is the second most common and fastest-growing neurodegenerative disease in the world [1]. It affects more than 10 million people worldwide, representing approximately 15% of all dementias, and impacts 1–2% of the global population over the age of 60 [2]. PD symptoms can be divided into two groups, motor symptoms that include bradykinesia, rigidity, and tremors; and non-motor symptoms including sleeping disorders, cognitive impairment, and sensory abnormalities [3]. The disease is characterised by dopaminergic neuronal loss in the substantia nigra pars compacta, a midbrain structure that plays a key role in motor control [4], as well as further impact upon other brain regions that lead to no non-motor symptoms. In most PD cases, symptoms do not appear until 60–80% of dopaminergic neurones have degenerated, making future treatment options limited without earlier diagnosis [5].

The pathological hallmark required for formal diagnosis is the development of insoluble protein inclusion bodies within neuronal cells known as Lewy bodies (LBs) (Fig. 1). LBs possess a complex composition formed of protein and lipid, in which the main proteinaceous component is alpha-synuclein (αS) [6]. Other neurodegenerative diseases collectively referred to as synucleinopathies are also characterised by αS aggregation and include multiple system atrophy (MSA) and dementia with Lewy bodies (DLB). The involvement of αS in PD is further supported by evidence showing that pathology is accelerated by duplications and triplications of the *SNCA* gene that encodes αS. This leads to increased concentrations of αS and consequently results in LB formation and the development of familial parkinsonism [7–9]. Furthermore, several point mutants within *SNCA* have been identified in early onset PD and related synucleinopathies (A30G [10]/P [11], E46K [12], H50Q [13, 14], G51D [15, 16], A53T [17]/E [18, 19]/V [20], E83Q [21]). These single-point mutations influence the precise distribution of oligomers and conformers and their rate of formation. In some cases, these mutations can accelerate the disease by several decades [22–25]. These amino acids are found at the interface of mature fibrils in many recent high-resolution structural studies obtained by CryoEM and ssNMR [26–28]. These fibrils may or may not hold clinical significance relative to early aggregates, but the location of early onset point mutations at the dimeric interface provides evidence that these mature structures may be weakened, or at least altered, leading to rapid formation and increased populations of toxic soluble and poorly defined low-n oligomers. Although the native function of αS is yet to be fully resolved, it is proposed to regulate the release of synaptic vesicles via interaction with lipid membranes [29]. Indeed, overexpression and consequent aggregation of αS within animal models is toxic, resulting in neurodegeneration that is further enhanced by the above point mutants [30]. The case for αS is therefore particularly compelling, but other proteins have also been implicated in PD. These include loss of function mutations within protein kinases encoded by *LRRK2* and *PINK1 genes*, and *PARK* that codes for E3 ligase Parkin [31]. These can interact with and impact upon αS and are described in other detailed reviews [32] [33].

Currently, there is no cure for PD. The main medication currently available is levodopa in combination with dopamine antagonists and monoamine oxidase-B (MAO-B) inhibitors, to prolong the benefits of levodopa [34]. Levodopa is a blood-brain barrier penetrant that is the precursor to catecholamine neurotransmitters (dopamine, noradrenaline, and adrenaline). Once inside the brain, it is converted to dopamine where it can substitute for decreased concentrations arising from loss of dopaminergic neurons to ease symptoms in which rigidity and bradykinesia are the most responsive. Other drug options include Amantadine to help with tremors, Catechol-O-methyl transferase (COMT) inhibitors to prevent COMT from deactivating levodopa, Anticholinergic drugs to reduce tremors by blocking the neurotransmitter acetylcholine, and Adenosine A2A receptor antagonists, which can work in a similar manner to levodopa to reduce symptoms during levodopa ‘off times’. However, all of the above are symptomatic drugs that have no impact on underlying PD pathology. Several reviews outline detailed therapeutic options under development for PD [35]. This review specifically focuses on peptide-based approaches under investigation, their current progress, and the realisation of peptide therapeutics capable of slowing or even stopping PD progression.

## Peptides as therapeutic agents

Peptides and peptidomimetics represent a rapidly progressing therapeutic field (for comprehensive reviews see [36–38]). Following the application of insulin in 1921, over 80 peptide drugs have since been approved, with more than 170 currently undergoing clinical trials [39]. Historically, small molecules have dominated the drug market, as they possess several beneficial features that include low synthetic cost, high biostability and bioavailability, as well as potential for membrane permeability, and oral administration. However, for signalling pathways and protein communication networks to operate, proteins must interact with each other. These protein-protein interactions (PPIs) are typically formed over large and very broad surface areas that typically lack the required pockets for small molecule intervention. Similarly, many PPIs are uniquely located within the cell, rendering them inaccessible to much larger biologics such as antibodies and larger proteins that have recently been successful in targeting extracellular PPIs and as immunotherapies.

In contrast, short peptides can bridge the gap between small molecules and larger biologics; they can provide high specificity to potently inhibit PPIs, while their natural amino acid composition results in reduced toxicity since they are degraded into naturally occurring components. Specificity also reduces unintended off-target effects that are frequently observed with small molecule drugs, further reducing the toxicity of peptide drugs. Short peptides also fall beneath the threshold for an immune repose, being below the required size for effective MHC class II presentation. Moreover, well-developed means of peptide production, namely solid-phase peptide synthesis coupled with peptide purification, culminates in an abundance of highly pure peptide free from cell-based contaminants, while keeping production costs low [36–38]. Caveats include limited uptake into cells and, more specifically for the purpose of this review, the blood-brain barrier, and oral bioavailability. However, these shortcomings are becoming increasingly well-understood, and traditional barriers to peptide therapeutics can now be circumvented. For example, techniques to improve drug-like properties for peptides include incorporation of D-amino acids, use of peptoids [40] (poly-N-substituted glycines; side-chains attached to the backbone nitrogen), beta-peptides [41] (side-chains attached to the beta-carbon two atoms from the carboxylate), other non-natural peptide backbones, non-natural amino acids including N-methylation [42], and sequence retro-inversion [43]. These approaches can be deployed to overcome drawbacks such as protecting the peptide from proteolytic degradation and facilitation of cell uptake.

Here we explore peptide and peptidomimetic approaches to directly target αS, and in particular those reported to impact upon αS aggregation and toxicity. Key features that will be discussed include how they were derived, if they are modified, where they bind, how much is reported as needed for inhibitory activity, impact upon αS-mediated cytotoxicity, and information regarding their precise mode of action. Given recent developments in this area, the related development of immunogenic peptides to trigger an antibody response designed to reduce αS aggregation is also briefly discussed. The peptide-based aggregation inhibitors can be broadly defined as rationally designed, library-derived, or derived from key components within the αS sequence that are thought to be important in instigating aggregation.

## Methodology to monitor aggregation and toxicity

Here we briefly discuss common approaches used to monitor amyloid formation and consequent toxicity, as well as approaches used to demonstrate toxicity associated with amyloid formation and the impact of inhibitors on either process. Since many different approaches are applied, we have discussed those most commonly used by experimentalists. Briefly, to monitor aggregation of amyloid sequences, Thioflavin-T (ThT) (fluorescence), or historically Congo red (absorbance) dyes are added to the samples [44]. Both intercalate with amyloid oligomers/fibrils and for ThT this results in an enhanced emission peak that can be monitored using either end-point analysis or preferably real-time aggregation profiles with/without the addition of inhibitors. However, ThT-based aggregation poorly defines the precise nature of the aggregates being monitored and is therefore frequently undertaken in parallel with other techniques [45]. For instance, circular dichroism experiments enable parallel monitoring of the global secondary structural content, and in particular, the conversion to the beta-sheet rich structure populated as oligomers and fibrils are formed. More detailed structural analysis of mature fibrils has exploded in recent years via the advent of cryoEM and solid state NMR based approaches (e.g. see [28] and refs within). However, these approaches can only solve structures of the much larger aggregates since these are accessible to the methodology. In contrast, low-n oligomers are likely to be of most significant clinical interest for early diagnosis and in disease prevention but they are unstable and transiently populated. Size-exclusion chromatography (SEC) and SDS-PAGE separation of early aggregates, or crosslinked samples that seek to stabilise them for detection (e.g. via PICUP or glutaraldehyde), can also be used to provide evidence for specific types of oligomer [46]. Electron microscopy or atomic force microscopy can also be used to provide a visual monitor of aggregates, or lack of, in the presence of inhibitors. For toxicity assays neuronal cell viability is typically monitored using 3-(4,5-Dimethylthiazol-2-yl)-2,5-diphenyltetrazolium (MTT), adenosine triphosphate (ATP), adenylate kinase, or lactate dehydrogenase (LDH) based cell viability assays, using neuronal or neuronal-like cells such as SHSY-5Y, PC12 cells, primary neurons, or more recently iPS cells from patient-derived fibroblasts (see [47] for an overview of cell death assays). There are a myriad of other experiments undertaken within the research articles discussed below, however, the above approaches cover a great many of those most frequently used to monitor amyloid aggregation and associated toxicity in neuronal culture.

## Targetable domains within αS

αS, along with β- and γ-synuclein is a member of the Synuclein protein family. The α- and β-synuclein proteins are found primarily in brain tissue, where they are located at presynaptic terminals. γ-Synuclein is located in the peripheral nervous system and retina, and is a tumour progression marker in breast cancer [48]. αS contains 140 amino acids (14.6 KDa) and exists in solution as an intrinsically disordered protein (IDP). However, there are several circumstances in which αS can assemble into well-defined secondary structures: ***(i)*** In an α-helical conformation that typically becomes populated by the presence of lipids [49, 50]. This structure is intricately linked to the native role αS plays in vesicle budding at the synaptic termini, and consequent synaptic transmission of dopamine. ***(ii)*** During aggregation into first oligomers, and later protofibrils and mature fibrils. During aggregation, αS adopts a β-strand rich amyloid conformation that is consistent with αS samples extracted from Lewy bodies [28]. If αS can be inhibited from forming these β- strand rich structures, it is therefore expected that its toxicity can be reduced.

**Fig. 1:**
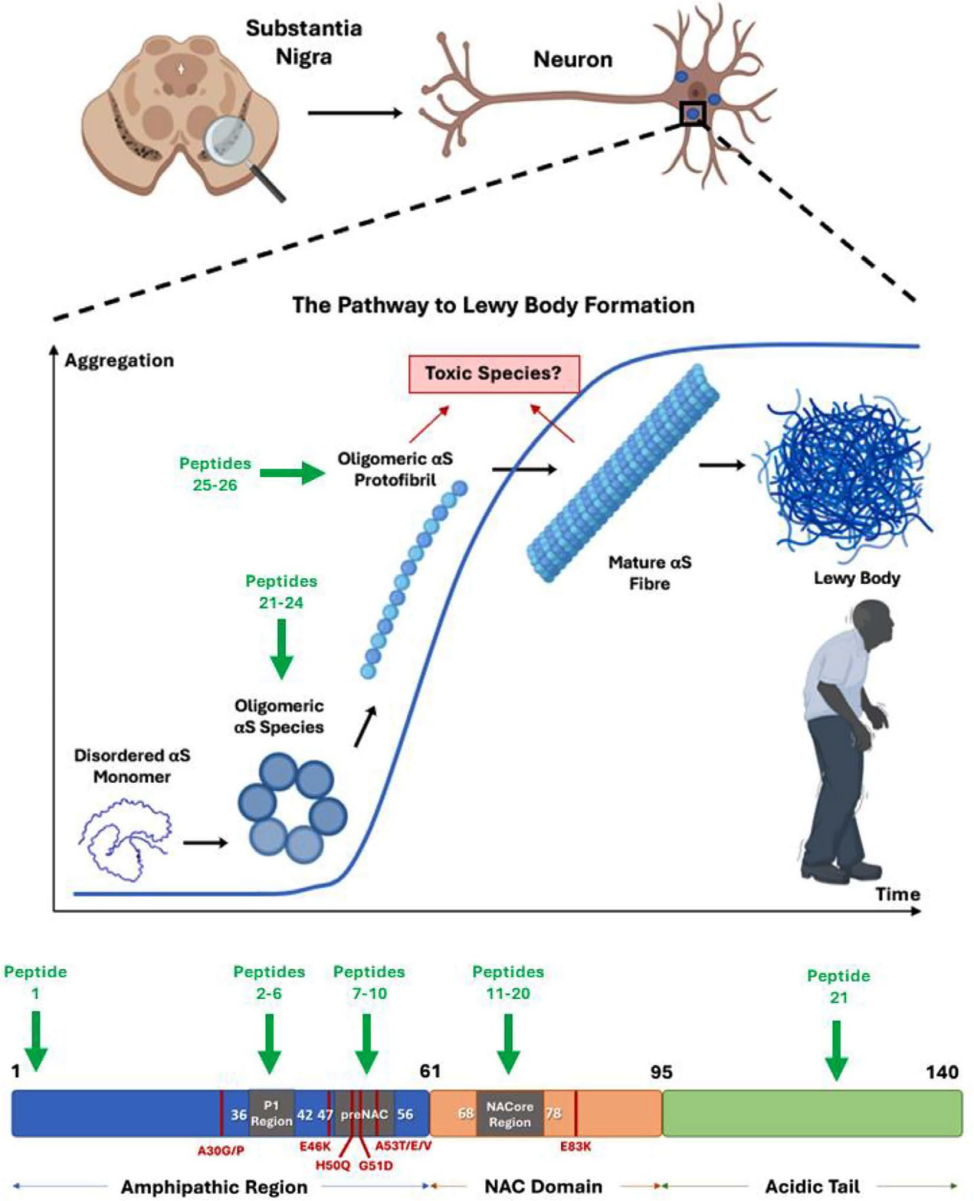
αS aggregation into Lewy bodies in PD and regions of interest. **Top**: In PD, alpha-synuclein (αS) is able to aggregate, via oligomers, into protofibrils, fibrils and Lewy bodies, leading to the death of dopaminergic neurons in the substantia nigra pars compacta region of the brain, which ultimately results in PD symptoms. Also shown is an aggregation profile in which the lag-phase, exponential phase and stationary phases are shown along with corresponding species of αS known to form. Figure created with BioRender.com. **Bottom**: the primary structure of αS is shown divided into three distinct regions. (1) Amphipathic N-terminal region (residues 1–60) containing seven imperfect repeats of the KTKEGV sequence and where most familial mutations linked to early-onset PD are found [62]. (2) NAC (non-amyloid component) domain, a central hydrophobic region (residues 61–95) involved in protein aggregation. (3) A negatively charged and proline-rich C-terminal region (residues 96–140). Point-mutations linked to early onset-PD (A30P/G, E46K, H50Q, G51D, A53T/E/V respectively) are shown in red for reference. Also shown are peptide sequence identifiers (green) corresponding to compounds in Table 1 and where within αS, and/or the aggregation pathway, that they are thought to target and impact

αS can be subdivided into three distinct domains: **(1)** an N-terminal amphipathic region (residues 1–60) that is key in lipid binding and in promotion of α-helicity; **(2)** a central hydrophobic component (residues 61–95) that is considered important for amyloid formation, confusingly referred to as the non-amyloid β-component (NAC) region; and **(3)** an acidic C-terminal tail (residues 96–140) that is poorly resolved in the majority of structural studies [50] (Fig. 1).

The N-terminal region (approximately residues 1–30) is known to adopt an α-helical conformation in the presence of lipids due to the amphipathic nature of imperfect repeats with the consensus sequence of KTKEGV. This repeating hexameric motif is found within lipid binding domains of related apolipoproteins and is the focus of several studies designed to target αS. The helical conformation exhibited by the N-terminus of αS has high homology with the class A_2_ helix, due to the arrangement of key Lys and Glu residues that flank a hydrophobic face [51]. The interaction of Lys residues with negatively charged head groups within the phospholipid membrane results in a high-affinity interaction and facilitates the packing of the hydrophobic face deep within the lipid bilayer, further stabilising the interaction. Moreover, the N-terminal region holds many point mutations linked to early onset of PD (A30G/P, E46K, H50Q, G51D/E, A53T/V/E; Fig. 1). Point mutations within a specific region, termed the preNAC region (^46^**E**GVV**HG**V**A**TVA^56^), may disrupt membrane interactions due to polarity clashes, precipitating the accumulation of αS while simultaneously modulating its ability to self-associate, since this region is located at the dimeric fibril interface of many high-resolution fibrillar structures (see [28] and refs within).

The central, hydrophobic, non-amyloid component (NAC) region of αS has been described as highly aggregation prone and contains one of the minimal sequences needed for aggregation, αS_71 − 82_, thought to be responsible for instigating aggregation of the full-length protein and therefore the focus of many early publications and attempts to inhibit αS aggregation [52]. Atomic resolution structural studies have identified that the NACore region αS_68 − 78_ can form symmetrical cross beta-sheet folds, stabilising peptides in the misfolded fibril in a β-sheet conformation [53]. αS_68 − 78_ (^68^GAVVTGVTAV^78^) [54] and related sequence αS_77 − 82_ (^77^VAQKTV^82^) [55] were previously noted to accelerate aggregation of this region. Deletion of the NAC region from αS results in a high resemblance of beta-synuclein (βS), a less neurotoxic synuclein, with some claims that it may also reduce and protect against αS neurotoxicity [56].

The αS C-terminus is proline rich, highly negatively charged, and able to bind multivalent cations. Importantly, it has been observed that αS-metal interactions can expedite αS aggregation rates [57]. The C-terminus is also noteworthy as a key region for post-translational modification. Four of the seven phosphorylation sites within αS are found in this region (Y125, S129, Y133, and Y135) [58]. Phosphorylation at other sites such as the recently reported T64 [59], but also at S129, is heavily linked to aggregation, with > 90% of αS extracted from Lewy bodies found to be S129 phosphorylated [60]. A physiological role for phosphorylation may be linked to the ability to coordinate metals, however the impact upon αS aggregation is poorly understood [61] and it is not determined if this modification occurs pre or post aggregation or prior to fibril formation.

## Targeting αS with peptides

The development of techniques to monitor aggregation and fibril formation such as ThT binding fluorescence assays, typically coupled with circular dichroism and Transmission Electron Microscopy (TEM), has facilitated the generation of many potent inhibitors of αS aggregation. For many other diseases, historically, small molecules have been pursued since these typically possess drug-like features such as bioavailability and cell penetrance that can be established in advance of inhibitor screens. Many adhere to Lipinski’s rule of 5, a set of lenient directives (e.g. LogP < 5, MW > 500 Da, fewer than 5 hydrogen bond donors) that can guide in establishing if a molecule is likely to be druglike [63]. Peptides are not typically Lipinski-like, but there is an increasing ability to modify them to circumvent barriers to their use as drugs. In the past decade, research into the application of peptide therapeutics to halt the progression of functional αS into harmful oligomeric species has grown rapidly. The routes by which functional peptide inhibitors are generated have also broadened. Classical methods of rational sequence and structure-based peptide designs have now expanded to include the screening of large peptide libraries to identify functional hits.

### Peptides derived from and targeting the N-terminus

The N-terminal region contains two main sequences of interest in the design of peptide inhibitors: the preNAC region, home to five of the common genetically linked mutations; and more recently, the P1 region (approximately residues 36–42) [62]. Yoshida et al. targeted the N-terminal region following previous studies from the group that had reported pyrroloquinoline quinone (PQQ) and epigallocatechin (EGCG) could be conjugated with αS via Lys sidechains to modify fibrilization [64]. Three sequences (αS_3 − 13_, αS_21 − 28_, αS_36 − 46_) were used to study the effect of incorporation of PQQ into small peptides. The most effective peptide, PQQ-αS_36 − 46_, reduced ThT fluorescence by > 60% in aggregation experiments, using a peptide:αS ratio of 10:1. Subsequent toxicity experiments showed a reduction of αS_119_ induced U2-OS cell toxicity by ~ 40 fold following the addition of PQQ-αS_36 − 46_. Little work has been performed since to characterise the mode of action for this peptide.

Another approach to target the αS N-terminus is to stabilise the alpha-helical form of the protein in which the first 30 residues or so are known to be important. To investigate this, Bavinton et al. rationally designed a peptidomimetic scaffold based on complementary contacts found within familial point-mutants. [65] An oligobenzamide backbone with the potential to form helix–mimic interactions like the helix-helix scaffold was tested for αS anti-aggregation activity. Mutational hotspots A53, H50, and E46 were targeted, and complementary side chains were incorporated onto the tribenzamide scaffold. The most effective peptidomimetic construct was found to stabilise the αS monomeric form and inhibit αS aggregation in the presence of lipid bilayers, with no apparent increase in ThT fluorescence over 60 h. However, the clinical value requires further evaluation in both in vitro and in vivo PD models to support the rationale behind the design of this peptide inhibitor. More recently Meade and co-workers used αS_1 − 25_ to prevent αS aggregation in a dose-dependent manner, offering further evidence for a potential mechanistic route to therapeutic intervention using this domain [66].

Horsley targeted the P1 master regulator region 36–42 determined by Doherty et al. using L- and D-isoforms of an N-terminal region targeting peptide (NTR-TP; GVLYVGS-Aib) as well as the heavily studied 77–82 region using a NAC targeting peptide (NAC-TP; VAQKTV-Aib) [67]. NAC-TP bears resemblance to Madine’s N-methylated peptide [55], with both peptides designed to adopt a beta-strand conformation. The C-terminal Aib group acts to prevent monomer addition to fibril ends. While NAC-TP D/L peptides were less effective at reducing αS aggregation; both NTR-TP D/L peptides reduced αS aggregation significantly (98% inhibited over 24 h at a ratio of 1:5 αS:peptide) and also solubilised fibrils (up to 78% for the NTR-TP D-peptide). NTR-TP-D was found to be effective in preventing αS primary nucleation and fibril elongation stages, and largely restoring cell viability in Caco-2 and Neuro-2 cytotoxicity assays.

Rezaeian and co-workers segmented proteins deposited in the PDB by searching those that adopt β structure and share similarity with αS regions involved in aggregation or contain PD associated mutations (αS_46 − 53 and_ αS_70 − 75_). [68] Next, β-strands adjacent to those sequences were tested for inhibition of αS aggregation. Two peptides, KISFRV and GQTYVLPG, were identified from RhoGDI and putative polyketide cyclase respectively, and shown to suppress aggregation in vitro, with KISVRV able to disrupt αS oligomers. Subsequently, an F4V change was implemented (KISVRV) to favour beta strand formation, with efficacy monitored via ThT fluorescence - with aggregation reduced between 80 and 90% at 1:1 after 6 days. The sequence was found to remove αS oligomers, as monitored via SEC, and the formation of a single monomeric peak was observed after four days of incubation. GQTYCLPG was less effective, with a peptide excess of 1:40 required to induce some inhibitory effect.

### Peptides derived from and targeting the NAC region

The hydrophobic NAC region (residues 61–95) is highly conserved between species and is amyloidogenic [52]. Numerous studies have demonstrated this region is involved in amyloid aggregation and it has been a major focus in studies to design peptides that target this region.

Using full-length αS as a template to create a library of overlapping 7-mer peptides, El-Agnaf et al. identified peptides targeting the amyloidogenic sequence αS_64 − 86_ [69]. Several short peptides incorporating αS_69 − 72_ were modified to include a solubilising component, RG/R, and GR residues, to the N- and C-terminal ends, respectively. These peptides were found to inhibit αS aggregation into both oligomeric and fibrillar species. The addition of an arginine-rich carrier to the ASI1 peptide, ASI1D, enabled membrane permeability to confer protective effects on iron-induced DNA damage and cell health in a cell line overexpressing mutant αS. However, one of these peptides was found to be ineffective as an aggregation inhibitor in a different study [70]. The protective effects of ASI1D were later confirmed in an oligodendroglial cell line, blocking the development of apoptotic markers caused by αS-induced aggregation [71]. Inconsistent effects of peptide inhibitors have historically conflated experimental difficulty, with differences in aggregation rates and morphologies reported even under the same incubation conditions [72].

Soon-Kim and co-workers studied mutations within the αS sequence that were observed to prevent aggregation from their previous study (e.g., V66S/P, T72P, V74E/G, T75P) [73]. The mutations were thought to be beta-breaking, therefore preventing fibril formation [74]. Small peptides (6–12 residues) were studied for each mutation. From an initial αS_68 − 77_ peptide (T72P-10mer) it was found that removal of the first four residues had no effect on its inhibition of aggregation. Similarly, removal of the first 4 residues from the N-terminus resulted in no inhibitory effect. The resulting αS_72 − 77_ ( T72P-6mer) was not only able to reduce the aggregation of αS by ~ 90% (1:5 excess peptide) but also remove mature fibrils. It was suggested that the peptide binds to monomeric forms, or early, low-n oligomers of αS. Marotta et al. then explored the O-GlcNAc modification at T72 and reported that the modified peptide behaved similarly to the T72P peptide studied by Soon-Kim [75].

Several other studies have explored the approach of short N-methylated peptides (shmeptides) targeting the NAC region, which have previously proven successful in inhibiting fibril formation and toxicity of amyloid beta [42, 76]. In a study by Bodles et al., N-methylation of NAC fragment αS_68 − 78_ led to disruption of fibril formation and αS associated toxicity [54]. Building on this idea, NMR was used for the first time for the rational design of peptide inhibitors based on the atomic structure of αS, identifying αS_77 − 82_ as a key region for aggregation [55]. This approach yielded an effective peptide based on αS_71 − 82_ with N-methylated C-terminal Val (VAQKTmV), corresponding to residues 71–82 which were found to constitute a self-recognition element within native αS [52]. This peptide reduced αS aggregation by up to 60% following 7 days at equimolar concentrations. The same peptide inhibitor identified by Madine et al. was found to be efficacious against fibrillogenesis and αS-induced toxicity in vitro in the presence of copper, a known accelerator of aggregation and toxicity [77]. However, N-methylation at other Val or Ala sites, promoted self-aggregation or aggregation, with careful placement of the N-methyl group required to confer the desired specificity of inhibition.

Recent advances in the understanding of structural interactions between the fibrils have facilitated the rational design of peptide inhibitors between dimer interfaces. Sangwan et al. also utilised the atomic NACore structure, that revealed a beta-strand interface, using the same NAC residues as Bodles et al. (aS_68 − 78_ [54]) as their design template. Theoretical peptides were generated using Rosetta-based computational modelling. Peptides were then scored based on binding energies, with four identified (S37, S61, S62, S71) that were found to inhibit amyloid formation in vitro [78]. Design requirements imposed an interruption to the beta-strand interface to provide a steric cap at fibril ends to prevent further elongation. All four retained the majority of the NACore sequence (_69_AVVTGVTAV_77_), with T72W introduced to provide a steric clash with the incoming β-sheet binding partner. None of the peptides were found to self-aggregate. The best two peptides, S61 and S62, contained the altered NACore inhibitory sequence (AVVWGVTAV), with the addition of a C-terminal TAT tag to increase solubility, prevent self-aggregation and improve cell penetration. Sequence scrambling resulted in the loss of the inhibitory effect, demonstrating sequence specificity for binding and inhibition, an idea later debated by Santos and co-workers, who found inhibitory effects of endogenous helical peptide LL-37 to be not based on sequence alone [79]. Although the proposed mechanism of action was related to the seeding pathways of αS, S61, a strongly performing inhibitor in in vitro experiments, performed poorly against seeding of MSA-derived fibrils [78]. This highlights the need for direct clinical relevance, which most studies currently lack. Both peptides, S62 and S71, are currently being developed as therapeutics; SLS-007 is an adeno-associated virus 1/2 vector being developed by Seelos Therapeutics that encodes both peptides.

### Peptides derived from other proteins

Liang et al. attempted to derive a peptide to stabilise monomeric αS [80]. Using a novel approach, they identified small ubiquitin-like modifier (SUMO) interacting motifs (SIMs) within αS with which SUMO can bind and mediate protein interactions critical to its physiological function [81, 82]. Using SUMO1 truncations Liang et al. identified a SUMO1-derived peptide (15–55) with high αS affinity. This peptide inhibited αS aggregation and suppressed toxicity in vitro and *in vivo.* However, an affinity for αS was found to be more favourable for one intact SIM than two, suggesting that the usefulness of this peptide as a widespread therapeutic may be limited.

Utilising a different approach to inhibit αS aggregation, Shaltiel-Karyo et al. derived a peptide based on βS [83], which shares highly conserved N- and C-terminal domains that can impact αS aggregation [84]. Using NMR, they identified the interaction of βS36 (GVLVGSKTR) with key αS residues, later defined as the P1 region. βS36 was found to inhibit αS aggregation by 80% (1:20 peptide excess). A retro-inverso analogue of βS-36 (RTKSGVYLVG) increased inhibition (> 98% at 1:20 peptide excess). This modified peptide was also more stable in mouse serum and able to penetrate SH-SY5Y cells in which αS was overexpressed [83]. Moreover, the βS36 retro inverso peptide could effectively reduce early and late-stage aggregation and improve locomotion in a PD fly model. Similarly, Wang and colleagues developed Tat-βS-degron as a βS derived peptide. The 24-mer peptide is comprised of three domains; a cell penetrating peptide Tat (GRKKRRQRRR), βS_36 − 45_ (RTKSGVYLVG), and the proteasomal targeting domain degron (RRRG), a peptide signal that is sufficient to direct its tagged proteins to proteasomes for degradation. The peptide was found to decrease αS aggregates and microglial activation in a pre-formed fibril model of spreading synucleinopathies in transgenic mice overexpressing human A53T αS. Moreover, Tat-βS-degron reduced αS levels and significantly decreased the Parkinsonian toxin-induced neuronal damage and motor impairment in a mouse toxicity model of PD.

Chemerovski-Glikman and co-workers studied a cyclic hexapeptide identified to assemble into supramolecular structures resembling amyloid, CP-2 (JWHSKL, J = norLeu). This peptide was able to bind the NAC region (and N-terminal region) of αS to reduce iron-induced toxicity in human neuronal cells overexpressing αS [85]. Self-assembled cyclic peptides CP-2, N-Me-CP-2, and CP-3 were synthesised, with CP-2 identified to both modulate αS aggregation by remodelling fibrils to non-toxic species, but also disaggregate pre-formed amyloid fibrils. N-Me-CP-2 and CP-3 were observed to be less active than CP-2 (CALCDPWW) in inhibiting the aggregation of αS. These cyclic peptides were found to accumulate in lysosomes or endosomes, suggesting neuronal cell penetration via endocytosis, a desirable trait relative to other peptides which typically require additional modification or appendage for such therapeutic benefit.

### Library-derived peptides

Kritzer and colleagues generated a head-to-tail cyclic peptide library using SICLOPPS and applied it to a yeast model of αS toxicity to recapitulate cellular PD pathology. From 5 million transformants the group identified two 8mer peptides, cyclic-peptide 1 and 2 (CP1 and CP2), that specifically reduced toxicity of expressed human αS, and prevented dopaminergic neuron loss in a *C.elegans* PD model [86].

In the absence of detailed structural information available at the time, in 2015 Cheruvara et al. deployed preNAC region αS_45 − 54_ as a design scaffold in which to build and screen a 210,000-member library in *E.coli* [44]. Since most early-onset point mutations are in this region (E46K, H50Q, G51D, A53T/V/E), it was deemed likely to be an important aggregation modulator and therefore a focal point for peptide aggregation inhibitor design. Subsequent high-resolution structural work has confirmed this region to be important in beta-strand formation at the dimeric interface of mature fibrils (see [28] and refs within). Cheruvara *et al.* assembled a library based on αS_45 − 54_ which contained the wild-type sequence, all known single-point mutant options found in early-onset PD known at the time, and a broad range of alternative conserved options. Intracellular toxicity rescue screening using a split DHFR Protein-fragment Complementation Assay (PCA) approach identified a potent inhibitor of aggregation and toxicity, 4554 W (KDGIVNGVKA). This was consequently found to function by inhibition of primary nucleation, revealing impact at the earliest stages of aggregation, inhibiting oligomer and subsequent fibril formation at the surface of lipid vesicles [87]. Later work utilising nuclear magnetic resonance (NMR) demonstrated this peptide exhibited its effect by binding low-n αS aggregates, to limit downstream aggregation and fibril maturation [88]. Deployment of Ala scanning next identified key residues required for activity while both downsizing the peptide and improving efficacy, resulting in 4654W(N6A) (DGIVAGVKA) [89]. Finally, PCA screening of the same previous 210,000-member library against five of the known αS single-point mutants associated with early-onset PD (to enhance stringency during screening) resulted in five peptides that shared many common features. All reduced aggregation of the respective point-mutant target, with several identified to be effective at reducing aggregation and toxicity in an SH-SY5Y line when incubated with other variants. Interestingly, the results demonstrated the downsized and optimised sequence 4654W(N6A) to be highly effective against not only WT αS_1 − 140_, but also several single-point mutant forms, and hence is a suitable baseline for further work toward a PD therapeutic [90].

In a related study, sequence deletions within the N-terminal region of full-length αS were shown to modulate aggregation. For instance, deletion of αS region ^36^GVLYVGS^42^ (P1) abolished aggregation. Further, P1, and the extended preNAC sequence, ^45^KEGVVHGVATVAE^57^ (P2), were shown to be required to promote fibril formation. Following from previous work that sought to identify sequence determinants of αS fibrillation, a library-based β-lactamase tripartite fusion construct in which αS was inserted between Blac fragments and probed for aggregation, identified mutations within the P1/P2 regions that impacted on the ability of αS to aggregate. These works collectively provide further sequence-based evidence for the role of this region in influencing aggregation lag-phase and consequent toxicity [62, 91].

Popova et al. screened a naïve peptide library of approximately one million members using a yeast-two-hybrid toxicity rescue approach to identify two synthetic peptides, K84s, and K102s, of 25 and 19 amino acids, respectively. These were found to inhibit αS oligomerization and aggregation at sub-stoichiometric molar ratios, with K84s showing some evidence of reducing αS aggregation in human neuroglioma (H4) cells [92]. Immunoblotting experiments suggest the peptides bind to low-n oligomers and not monomeric αS, however, further analysis is required to identify the specific region of αS that the peptides interact with.

Prirhaghi et al. utilised peptide arrays to divert aggregation from fibrils to amorphous aggregates with reduced β-sheet content and increased random coil. However, they did not reduce cytotoxicity. However, a non–self-aggregating (p216) peptide derived from the N-terminus of penetratin showed 12-fold higher binding affinity to oligomers than to αS monomers, was not toxic to SH-SY5Y cells, and reduced αS cytotoxicity by about 20%. [93].

Nim et al. performed a high throughput proteome-wide peptide screen to identify PPI inhibitors that restored endolysosomal function to reduce αS oligomer levels and associated cytotoxicity and DA neuron loss in *C. elegans* and preclinical PD rat models [94]. The lentiviral library consisted of 50,549 7-mer peptides enriched in PPI motifs. For selection, HEK293T cells were infected in a pooled format, with each peptide amino acid sequence serving as its own barcode. Puromycin selection was then used to screen the GFP-tagged library for peptides that reduced αS-mediated cytotoxicity or αS-oligomers in parallel, therefore achieving cell survival and enabling peptide identification. The most potent inhibitor identified, PDpep1.3, was found to disrupt the interaction between the αS C-terminus and CHMP2B, an Endosomal Sorting Complex Required for Transport-III (ESCRT-III) component, which plays an important role in maintaining cellular homeostasis. This interference restores ESCRT function, and therefore the peptide enhanced clearance of the overexpressed αS.

Most recently, Suga and coworkers utilised a form of mRNA display known as the random non-standard Peptides Integrated Discovery (RaPID) system. [95]. This puromycin-ligated mRNA library was constructed to encode macrocyclic peptides with N-chloroacetyl-Tyrosine as an initiator followed by a random peptide region, followed by Cys, and ending with a short linker peptide; such that upon mRNA translation, the chloroacetyl group on the N-terminus of the library is spontaneously cyclized with the downstream cysteine to form thioether-macrocyclic peptides. The approach identified a macrocyclic peptide, BD1, that interacted with immobilized αS to inhibit fibril formation. Upon further improving BD1 solubility, the peptide was found to suppress co-aggregation with αS fibrils and displayed more effective kinetic inhibition without changing fibril morphology. BD1 and derivatives were studied and found to function by binding to fibril ends, thereby preventing fibril elongation.

### Helical peptides derived from bacterial protein PSMα3 and human protein LL-37

Phenol soluble modulin α3 (PSMα3) is a bacterial extracellular peptide that is secreted to provide a defence against an immune response by recruiting, activating and subsequently lysing human neutrophils [96] and is of interest given the potential role of the gut microbiome in initiating PD pathology. This 22-residue peptide exists in a stable, α-helical conformation. Santos et al. tested the peptide and showed that it does not target monomeric αS, but rather interacts with toxic ‘Type-B’ oligomers and αS fibrils [97]. ThT aggregation studies show the PSM3α peptide inhibits aggregation by 90% at equimolar concentrations between PSM3α and αS after 32 h. This bacterial helical peptide with cationic and amphipathic properties was found to inhibit αS aggregation with high affinity and selectivity and abrogate oligomer-induced damage in vitro [79]. The same group also studied human antimicrobial peptide LL-37, a peptide that is part of the innate immune system. It is formed from C-terminal cleavage by kallikreins or proteinase 3 of precursor hCAP18, resulting in the only cathelicidin derived antimicrobial peptide found in humans [98]. This 37-mer amphipathic peptide becomes excreted at sites of infection and primarily exists in an alpha-helical conformation [99]. LL-37 also preferentially binds toxic Type-B oligomers and fibrils. Following a 32-hour incubation, LL-37 was shown to reduce aggregation by up to 80% using a 2-fold excess of αS, with binding characteristics found to be similar to PSM3α. In more recent work, LL-37 was also found to be implicated in AD, where it may promote disease by triggering membrane translocation and the activation of the chloride channel CLIC1, ultimately inducing microglial hyperactivation [100]. Further research is required to determine the potential of LL-37 as a PD therapeutic.

### Peptides that trigger an immune response to αS

Following the discovery that αS can transmit from cell to cell within the brain [101] [102], Mandler et al. studied possible treatments to target extracellular αS [103]. Phage display was employed to screen a large library of short peptide sequences based on an αS_110 − 130_ C-terminal epitope. The peptides were then tested against monoclonal antibodies known to specifically bind to the C-terminal region of αS [104, 105]. Seven peptide sequences were studied, of which five were observed to induce an immune response to αS in mice models. Importantly, these peptides, termed AFF 1–5 specifically recognised αS, and not the βS protein. The AFF 1 peptide, 8 residues in length, was then studied further as this specifically induced a response to human αS. The peptide was shown to stimulate the desired B-cell antibody response, whilst evading the autoimmune T-cell response due to its small size, resulting in the reduction of oligomeric αS in PDGF- αS tg mice models. These immunogenic peptides, termed Affitopes (specifically PD01A and PD03A) are currently waiting for phase 2 trials to begin.

**Table 1:** Peptide derivates designed to directly impact on aggregation and toxicity of αS. The peptide identifier number listed matches to numbers shown in Fig. 1 (green) to indicate for those known where they bind within αS and where within the aggregation profile they are thought to impact

		Peptide Antagonists of αS Aggregation / Toxicity				
Peptide Name	Length	Sequence	Target αS Region/Species	Effect	Peptide Identifier	Reference
aS_1 − 25_	25	MDVFMKGLSKAKEGVVAAAEKTKQG	N-terminal lipid binding	< 80% reduction in lipid-induced ThT Fluorescence at 1:10 (αS:Peptide)	1	Meade 2023 [66]
Retro-inverso βS36	10	RTKSGVYLVG	36–45 in βSyn. (highly conserved in αS)	> 98% reduction in ThT Fluorescence at 1:20 (αS:Peptide) 2.Drospsphila model overexpressing A53T αS mutant recovered 86% locomotion following peptide treatment.	2	Shaltiel-Karyo 2010 [83]
αS_36 − 46_-PQQ	11	GVLYVGSKTKE	36–42, p1 region	> 60% reduction in ThT Fluorescence at 1:10 (αS:Peptide) 2. Reduced toxicity of aS_119_ mutant by ~ 40% in U2-OS cells.	3	Yoshida 2013 [106]
Tat-βsyn-degron	25	YGRKKRRQRRRRTKSGVYLVGRRRG	36–45 in βSyn. (Highly conserved in αS)	Reduced αS levels over 24 h in M83 transgenic mice overexpressing A53T αS.	4	Jin 2021 [107]
NTR-TP-L	7	GVLYVGS-Aib	p1 region	98% reduction in ThT Fluorescence at 1:5 (αS:Peptide) 2. Restored 84% cell viability in Caco-2 MTT Cytotoxicity assays.	5	Horsley 2022 [67]
NTR-TP-D	7	GVLYVGS-Aib	p1 region	98% reduction in ThT Fluorescence at 1:5 (αS:Peptide) 2. Restored 88% cell viability in Caco-2 MTT Cytotoxicity assays.	6	Horsley 2022 [67]
4554 W	10	KDGIVNGVKA	preNAC (αS_45 − 54_)	1.>90% reduction in ThT Fluorescence at 1:1 (αS:Peptide) 2. Restored 80% cell viability in PC12 MTT Cytotoxicity assays.	7	Cheruvara 2015 [44]
4654 W(N6A)	9	DGIVAGVKA	preNAC	1. >98% reduction in ThT Fluorescence at 1:10 (αS:Peptide) 2. Restored 100% cell viability in SH-SY5Y MTT Cytotoxicity assays.	8	Meade 2021 [89]
GQTYVLPG	8	GQTYVLPG	46–53 preNAC	< 90% reduction in ThT Fluorescence at 1:40 (αS:Peptide)	9	Rezaeian 2017 [68]
CP-2	6	JWHSKL (Cyclic) J = Norleucine	NAC and N-Terminal	1. ~70% reduction in ThT Fluorescence at 1:1 (αS:Peptide) 2. Improved cell viability by ~ 20% in PC12 MTT Cytotoxicity	10	Chemerovski-Glikman 2016 [85]
S37	15	GAVVWGVTAVKKKKK	68–78 NACore	1. >90% reduction in ThT Fluorescence at 1:10 (αS:Peptide) 2. Reducing αS seeding by ~ 60%	11	Sangwan 2020 [78]
S61	22	GAVVWGVTAVGRKKRRQRRRPQ	68–78 NACore	1. >80% reduction in ThT Fluorescence at 1:2 (αS:Peptide) 2. Reducing αS seeding by ~ 30%	12	Sangwan 2020 [78]
S62	24	GAVVWGVTAVKKGRKKRRQRRRPQ	68–78 NACore	1. >90% reduction in ThT Fluorescence at 1:1 (αS:Peptide) 2. Reducing αS seeding by ~ 80%	13	Sangwan 2020 [78]
S71	22	YGRKKRRQRRRAVVT (nme-Gly)VTAVAE	68–78 NACore	1. >70% reduction in ThT Fluorescence at 1:10 (αS:Peptide) 2. Reducing αS seeding by ~ 60%	14	Sangwan 2020 [78]
KISVRV	6	KISVRV	70–75 NAC	1. <90% reduction in ThT Fluorescence at 1:5 (αS:Peptide) 2. Removed oligomer peak from SEC following 4-day incubation.	15	Rezaeian 2017 [68]
T72P-6mer	6	PGVTAV	72–77 NAC	< 90% reduction in ThT Fluorescence at 1:5 (αS:Peptide)	16	Soon Kim 2009 [74]
71–76	6	mVTGVTA VTGmVTA	NAC region	No inhibitory effect	17	Madine 2008 [55]
77–82	6	VAQKTmV VmAQKTV	NAC region	1. 60% reduction in ThT Fluorescence at 1:1 (αS:Peptide)	18	Madine 2008 [55]
NAC-TP-L	6	VAQKTV-Aib	NAC region	No inhibitory effect	19	Horsley 2022 [67]
NAC-TP-D	6	VAQKTV-Aib	NAC region	50% reduction in ThT Fluorescence at 1:5 (αS:Peptide) 2. Restored ~ 70% cell viability in Caco-2 MTT Cytotoxicity assays.	20	Horsley 2022 [67]
PDpep1.3	12	DEEIERQLKALG	C-terminus	PDpep1.3 reduced pS129 αS accumulation in striatum of rat model.	21	Nim 2023 [94]
K84s	25	FLVWGCLRGSAIGECVVHGGPPSRH	Oligomers	1. >75% reduction in ThT Fluorescence at 1:1 (αS:Peptide) 2. Reduced αS inclusion formation by ~ 20% in human neuroglioma cells (H4)	22	Popova 2021 [92]
K102s	19	FLKRWARSTRWGTASCGGS	Oligomers	> 80% reduction in ThT Fluorescence at 1:1 (αS:Peptide)	23	Popova 2021 [92]
p216	11	WFQNRRMKWKK	Oligomers	Improved cell viability by ~ 20% in SH-SY5Y MTT Cytotoxicity assays.	24	Pirhaghi 2022 [93]
PSMα3	22	MEFVAKLFKFFKDLLGKFLGNN	Type B [97] oligomers Fibrils	1. >90% reduction in ThT Fluorescence at 1:1 (αS:Peptide) 2. K_d_ of 3.62nM for toxic type B* oligomers	25	Santos 2021 [79]
LL-37	37	LLGDFFRKSKEKIGKEFKRIVQRIKDFLRNLVPRTES	Type B [97] oligomers Fibrils	1. >80% reduction in ThT Fluorescence at 2:1 (αS:Peptide) 2. K_d_ of 5.14nM for toxic type B* oligomers	26	Santos 2021 [79]
CP1	8	CPWCSTRV	Unknown	Reduced neurodegeneration in C.elegans by 74%	27	Kritzer 2009 [86]
CP2	8	CALCDPWW	Unknown	Reduced neurodegeneration in C.elegans by 74%	28	Kritzer 2009 [86]
BD1	16	^D^YSGLIKWTTALLRTYC(D^Y^ = N-chloroacetyl-D-tyrosine, which cyclises with downstream cys to form a thioether-macrocycle)	Unknown	> 95% reduction in ThT Fluorescence at 1:1 (αS:Peptide)	29	Ikenoue 2023 [95]

### Conclusion and future directions

This review has discussed several peptide-based strategies against αS aggregation and/or toxicity. This has included peptides based on distinct regions of αS, the N-terminal region, including the preNAC region, the NAC region and the C-terminal domain, with peptides derived from residues within these regions or alternative sources. There is a large and growing body of literature demonstrating direct inhibition of αS aggregation and/or toxicity with differences in their design and mechanisms. However, inhibiting aggregation comes with the caveat that selective targeting of one specific stage of aggregation, may result in toxic species becoming even more populated. Therefore, stabilising the monomeric IDP, or potentially the helical form of the protein to block aggregation upstream of misfolding is perhaps still the most compelling approach. However, the multitude of different conformations that αS can exhibit poses a major challenge for the design of αS aggregation modulators. Since αS is natively an IDP in solution, bearing little to no ordered structure, rational design of compounds to stabilise the monomeric, non-toxic conformation is difficult. However, αS can adopt a helical conformation in the presence of lipids, and research presented in this review suggests that stabilising this form could be achievable [65, 66, 79, 80]. This would support either camp of thought around whether PD pathology is driven by a proteinopathy (toxic accrual of αS aggregates – gain of function) or a proteinopenia (loss of αS native function). Perhaps most importantly, an ongoing concern for the future of αS directed therapies is a lack of early diagnostic tools for PD. Therefore, the future clinical efficacy of some of the discussed peptides will need to be coupled with effective diagnostics at the prodromal or preferably even earlier stages of the disease where a therapeutic can be administered before significant dopaminergic loss. Work in this area is however continuing to develop, with recent αS seed amplification assay (SAA) studies demonstrating that low abundancy αS can be detected within CSF [108]. The SAA assay was also able to classify patients with PD with high sensitivity and specificity while providing information about molecular heterogeneity. The approach harbours significant potential to detect prodromal individuals prior to diagnosis to identify pathologically defined subgroups of people with Parkinson’s disease, and to establish biomarker-defined at-risk cohorts. Another area requiring attention is that although we now have a large pool of peptides that show efficacy (Table 1), it is difficult to directly compare attributes, since they have been characterised under different conditions, concentrations, and using many different methodologies. It would be prudent at this point to consolidate the efforts thus far, by independently testing peptides in parallel using a range of standardised techniques to determine those most suitable for a particular application. This effort would also spotlight those peptides most likely to translate into functional therapeutics.

Although attempts to derive peptide-inhibitors of αS aggregation is an active area of research, the field may want to consider the techniques used to identify novel inhibitors of aggregation. Utilising the growing pool of information arising from atomic resolution structures of αS polymorphs [26–28] and evaluating the physicochemical traits of both the protein and potential peptides could maximise the chances of identifying effective PD therapeutics. In the absence of diagnostic tools to identify early stages of PD progression, a mixture of inhibitors with differing mechanisms to recognise different aggregate populations may represent the most effective strategy. Most notably, peptide-based strategies have also shown promise as potential diagnostic tools able to discriminate between native αS and its pathogenic species [79] [109]. Therefore, the design of peptide inhibitors may also facilitate earlier PD detection in addition to enhanced therapeutic value and clinical effectiveness.

## Data Availability

Not applicable.

## Abbreviations

αS: Alpha-synuclein the major constituent of Lewy bodies and a pathogenic hallmark of all synucleinopathathies
PD: Parkinson’s disease
DLB: dementia with Lewy bodies
MSA: multiple system atrophy
SNCA: SyNuClein Alpha gene that codes for the αS protein
